# Aerobic glycolysis of vascular endothelial cells: a novel perspective in cancer therapy

**DOI:** 10.1007/s11033-024-09588-1

**Published:** 2024-06-01

**Authors:** Shenhao Xu, Jiahao Liao, Bing Liu, Cheng Zhang, Xin Xu

**Affiliations:** 1 Department of urology, the Fourth Affiliated Hospital of School of Medicine, and International School of Medicine, International Institutes of Medicine, Zhejiang University, Yiwu, 322000 China; 2 The First Affiliated Hospital, Zhejiang University School of Medicine, Hangzhou, 310000 China

**Keywords:** Vascular endothelial cells, Aerobic glycolysis, Cancer, Therapy

## Abstract

Vascular endothelial cells (ECs) are monolayers of cells arranged in the inner walls of blood vessels. Under normal physiological conditions, ECs play an essential role in angiogenesis, homeostasis and immune response. Emerging evidence suggests that abnormalities in EC metabolism, especially aerobic glycolysis, are associated with the initiation and progression of various diseases, including multiple cancers. In this review, we discuss the differences in aerobic glycolysis of vascular ECs under normal and pathological conditions, focusing on the recent research progress of aerobic glycolysis in tumor vascular ECs and potential strategies for cancer therapy.

## Introduction

Endothelial cells (ECs) form a monolayer of cells arranged along the inner lining of blood vessels. Under normal physiological conditions, ECs play a pivotal role in maintaining oxygen and nutrient supply to all bodily tissues [1]. ECs are critical for upholding the internal environment, its homeostasis, and immune function. In recent years, research on EC metabolism has gained significant momentum. A growing body of evidence has shown that EC metabolism undergoes significant alterations under various pathological conditions, profoundly impacting disease onset and progression. As an crucial link in cellular energy metabolism, aerobic glycolysis plays a remarkable role in EC activities and could offer novel targets for disease treatment. This paper aims to explore recent advances in glycolysis in ECs under normal and pathological conditions, with a particular emphasis on human cancers.

## The function of ECs in physiological state

### Angiogenesis

ECs are typically quiescent in healthy state but can be rapidly activated in response to pathological changes, facilitating the delivery of oxygen and nutrients to hypoxic tissues through the formation of new blood vessels, a process known as angiogenesis.

Angiogenesis is accomplished through the interaction among three distinct EC subtypes: tip cells, stalk cells, and phalanx cells, each fulfilling a specific role in the process. Neovascularization commences with the migration of tip cells, succeeded by the proliferation of stalk cells, which generate new vascular sprouts. Subsequently, phalanx cells, characterized by a nonproliferating phenotype, continue to align established vessels, facilitating the formation of mature vessels with the functions of regulating vascular homeostasis and establishing endothelial barrier [2].

The key cytokine that dynamically regulates the differentiation of ECs into one of these subtypes is vascular endothelial growth factor (VEGF), which binds to VEGF receptor 2 (VEGFR2) on ECs and induces their differentiation into tip cells. Tip cells express delta-like ligand 4 (DLL4), which binds to Notch receptors on neighboring ECs, initiating their differentiation into stalk cells. In stalk cells, DLL4 signaling induces cleavage of the Notch intracellular structural domain (NICD), which then induces the cell to stimulating the expression of produce VEGF receptor 1 (VEGFR1). VEGFR1 is substantially less sensitive to VEGF compared to VEGFR2, further ensuring that ECs adjacent to tip cells become stalk cells [3].

This seemingly strict interaction between tip and stalk cells is actually dynamic, and this dynamic competition ensures that cells with the highest VEGFR2/VEGFR1 ratio become tip cells [4].

### Maintaining internal environmental and its homeostasis

ECs serve as crucial intermediaries between human blood circulation and organ tissues, playing an indispensable role in the regulation of body functions. These functions include maintaining coagulation, regulating blood pressure, and facilitating the exchange of substances both within and outside of blood vessels, with particular significance in the regulation of coagulation. For instance, ECs regulate blood pressure and maintain the balance between coagulation and anticoagulation in blood flow by releasing vasodilatory substances such as prostaglandin I2 (PGI2) and nitric oxide (NO), as well as vasoconstrictive substances like endothelin 1 (ET-1) and angiotensin II (AngII) [5]. Moreover, ECs can produce nitric oxide synthase (NOS) in response to hormonal and chemical signal stimulation, and NO synthesized by endothelial NOS (eNOS) regulates peripheral vascular tone by stimulating NO-sensitive guanylate cyclase, which plays a vital role in maintaining vascular homeostasis and controlling blood pressure [6].

### Immune response

As the type of cells that are in direct contact with the circulation, ECs are pivotal participants and regulators of the inflammatory response. Their recognition and response to substances like invading circulating microorganisms are crucial for activating the body’s early immune system [7]. For instance, ECs express Nod1 to trigger the release of the inflammatory factor IL-8 [8]. There is also an inextricable link between the inflammatory response and the angiogenic process. In chronic inflammation, ECs can respond to angiogenic factors like VEGFA to sustain inflammatory angiogenesis [7].

## EC Metabolism under physiological conditions

Glucose, fatty acids (FA) and amino acids (AA) are the three primary substrates for adenosine triphosphate (ATP) and biomass production in ECs, and their roles have been extensively studied and summarized. The following section will summarize the role of EC metabolism in maintaining endothelial function and disease pathogenesis, with a specific focus on glycolysis in tumor vascular ECs.

### The glycolysis and bypasses of ECs

Under physiological conditions, glycolysis serves as the primary mode of energy production in ECs, exhibiting a higher proportion compared to other cells in the body. Similar to many cancer cells, up to 85% of ATP is produced by glycolysis in ECs. This phenomenon can be attributed to two possible reasons. Firstly, ECs protect themselves from oxidative stress by keeping reactive oxygen species (ROS) at a low level. Secondly, ECs would like to increase the oxygen supply to their surrounding tissues by means of anaerobic metabolism [9]. It has been demonstrated that when exposed to a low glucose environment or when glucose is competed by 2-deoxy-d-glucose (2-DG), a structural analogue of glucose, for the glycolytic process, ECs trigger ROS-induced autophagy, suggesting that ECs reduce oxygen dependence by preferentially utilizing glycolytic capacity, and thereby decrease ROS production [9, 10]. Additionally, the angiogenesis process in hypoxic tissues demands a faster rate of ATP production through glycolysis to expedite the re-establishment of blood circulation at hypoxic sites. This necessity also contributes to the high glycolytic rate observed in ECs.

In the process of angiogenesis, the initiation of the glycolytic pathway first involves the uptake of glucose into ECs via the membrane-bound glucose transporter protein (GLUT) [11]. The glucose-based glycolytic pathway is regulated by three key enzymes: phosphofructokinase-1 (PFK1), hexokinase 2 (HK2) and pyruvate kinase (PK).

PFK1 is an important rate-limiting enzyme in the glycolytic pathway, converting fructose-6-phosphate (F6P) into fructose-1,6-bisphosphate (F1,6P2). 6-phosphofructo-2-kinase/fructose-2,6bisphosphatase3 (PFKFB3), an efficient glycolytic activator, can variably activate PFK1 by producing fructose-2,6-bisphosphate (F2,6P2). PFKFB3 not only regulates the proliferation of ECs but also controls the formation and directional migration of filopodium and lamellipodium. Therefore, the deletion of PFKFB3 impairs the angiogenic function of ECs [12]. In contrast, overexpression of PFKFB3 leads to elevatedglycolysis levels in ECs exhibiting a pro-tip cell phenotype. It even inhibits Notch1 signaling in pro-stalk cells during retinal vascular development, further underscoring the importance of cellular glycolysis levels in ECs in determining the tip cell phenotype [12]. As previously previously, VEGF operates through the Notch signaling pathway to ensure that cells with the highest VEGFR2/VEGFR1 ratio become the tip cells. Similarly, it is reported that stalk cells can display higher glycolytic activity when expressing higher levels of PFKFB3, effectively taking the place of tip cells [12].

HK2 is another glycolytic rate-limiting enzyme responsible for catalyzing the initial step of glycolysis in ECs, which involves the phosphorylation of glucose in ECs to glucose-6-phosphate (G6P). Increasing evidence suggests that HK2 is upregulated in various types of tumors, correlating with enhanced aerobic glycolysis.The upregulation of HK2 expression can be attributed to fibroblast growth factor (FGF), which enhances the expression of MYC, one of the most frequently dysregulated driver genes in human cancers. Although MYC was previously deemed “undruggable” due to the lack of a suitable pocket for high-affinity binding of low-molecular-weight inhibitors, recent preclinical trials have shown significant anticancer effects of some MYC-targeted inhibitors. This suggests that such anticancer effects may also arise from the inhibition of aerobic glycolysis in tumor vascular ECs [13]. For example, it has recently been found that OTUB1 blocks MYC protein degradation through deubiquitination, thereby promoting HK2-mediated glycolysis and the development of breast cancer [14].

PK is also a glycolytic rate-limiting enzyme that converts phosphoenolpyruvate (PEP) to pyruvate. PKM2, one of the four tissue-specific isoforms of PK, exists as a dimer or tetramer in normal, malignant, and embryonic cells. The affinity of dimeric PKM2 for PEP is lower than that of tetrameric PKM2, resulting in less conversion to pyruvate, which inhibits glycolysis and leads glucose more into the glycolytic bypass for biomass synthesis. In highly proliferative endothelial cells, PKM2 tends to shift towards its dimeric form to promote biomass synthesis, aligning with their heightened proliferative properties [15–17]. As a glycolytic rate-limiting enzyme, silencing PKM2 in ECs inhibits neovascularization. Furthermore, Protein JMJD8 has been identified as associated with PKM2 to regulate the angiogenic process. Knocking down JMJD8 inhibits EC metabolism and angiogenesis, although the exact mechanism by which JMJD8 regulates PKM2 remains uncertain [18].

Several environmental factors and signaling proteins have been identified that can influence the glycolytic level and angiogenic capacity of ECs by regulating the three key enzymes mentioned above and their regulators. For example, mechanical signaling in the circulation can also affect angiogenesis in a metabolism-dependent manner, as evidenced by the activation of Krüppel-like factor 2 (KLF2) in response to laminar shear stress, which inhibits PFKFB3, HK2, and several other glycolytic genes [19]. The FOXO family is highly enriched in ECs. The transcription factor FOXO1 was found to inhibit glycolysis in ECs by suppressing MYC and PFKFB3 levels [20, 21]. Additionally, FOXO1 exhibits environment-dependent activity, promoting the germination and migration of lymphatic ECs by upregulating the purinergic receptor P2RY1 upon exposure to ATP [20]. Meanwhile, impairment of vascular growth and germination function was also observed after silencing PFKFB3 and HK2 in animal models, further suggesting the essential role of glycolysisl for ECs and angiogenesis in vivo [12, 22]. Furthermore, the availability of sufficient glucose in the microenvironment for uptake plays a decisive role in the level of glycolysis of ECs. Glycogen synthesis in ECs increases with sufficient glucose supply, leading to accumulation of intracellular glycogen, while deprivation of glucose causes the inhibition of glycogen phosphorylase (GP), which catalyzes glycogenolysis in ECs, impairing the ability of ECs to migrate and survive [23]. This evidence suggests that ECs utilize glycogen as reserve energy when carrying out angiogenic pro-cesses in a glucose-deficient environment.

The pentose phosphate pathway (PPP) is a bypass of glycolysis that facilitates the synthesis of raw nucleotide material and the homeostasis of redox balance by diverting G6P generated from glucose to produce nicotinamide adenine dinucleotide phosphate (NADPH) and ribose 5-phosphate (R5P). NADPH, in turn, can regenerate a common cellular antioxidant, reduced glutathione [24–26]. Additionally, NADPH also participates in fatty acid and NO production. The NADPH generated by the pentose phosphate pathway is directly involved in fatty acid synthesis, thus linking the PPP of ECs to fatty acid metabolism. Moreover, NO promotes the angiogenic activity of ECs [27]. Glucose 6-phosphate dehydrogenase (G6PD) is one of the rate-limiting enzymes of PPP. Overexpression of G6PD stimulates EC proliferation and migration by boosting NO and NADPH production. Another rate-limiting enzyme of PPP in the reversible non-oxidative pathway is transketolase. Inhibition of either of these two rate-limiting enzymes has been shown to significantly suppress EC survival [23, 28].

Another bypass of glycolysis is known as the hexosamine biosynthetic pathway (HBP). Although it contributes to a relatively small proportion of glucose metabolism, HBP is closely linked to post-translational protein modifications via glycosylation, including N-glycosylation and O-glycosylation. .N-glycosylation enhances the stability, membrane expression, and signaling activity of VEGFR2, while O-glycosylation influences interactions related to NOTCH signaling ligands. Consequently, HBP is implicated in determining the apical cell phenotype of ECs, underscoring its unique role in EC functions [29–32]. Further investigations are warranted to elucidate the mechanisms by which HBP impacts angiogenesis.

### Other metabolic pathways of ECs

#### FA metabolism of ECs

Generally, mitochondria are the primary site of ATP production, and acetyl coenzyme A derived from fatty acids FA is utilized in the tricarboxylic acid (TCA) cycle to main-tain ATP production. While in proliferating endothelial cells (PECs), the main function of mitochondria is biosynthesis. The dependence of dNTP synthesis in PECs on the carbon source in fatty acids is now well established. FA is metabolized to acetyl coenzyme A to maintain the TCA cycle. In addition to the production of ATP, the TCA cycle provides precursors for the synthesis of dNTP necessary for proliferation, thereby promoting DNA synthesis and thus aiding PECs proliferation [33]. Furthermore, surprisingly, quiescent endothelial cells (QECs) regenerate NADPH for redox homeostasis by enhancing FAO levels [34].

Carnitine palmitoyltransferase 1a (CPT1a) is a rate-limiting enzyme in the FA metabolic pathway that transfers FA into mitochondria. It has been discovered that CPT1a deficiency not only leads to attenuated proliferation of PECs and defective neovascularization in vitro and in vivo, but also promotes QECs dysfunction by increasing endothelial oxidative stress. Supplementation with acetyl coenzyme A precursors, which promote the tricarboxylic acid (TCA) cycle, restored cellular dNTP levels and alleviated oxidative stress-induced EC dysfunction in CPT1a-deficient ECs. Neither glucose nor glutamine metabolism could compensate for this metabolic defect of Fatty acid oxidation (FAO) [33, 34]. These findings indicates that ECs primarily utilize fatty acid-derived carbon for biosynthesis rather than energy generation through FA metabolism. This metabolic pathway supports cell proliferation during angiogenesis and facilitates the regeneration of NADPH to maintain cellular redox balance.

#### Glutamine metabolism of ECs

Glutamine, the most abundant non-essential amino acid (NEAA) in circulation, plays a crucial role in maintaining proliferation and vasodilation in ECs through its metabolism. In ECs, approximately 30% of the carbon source for the TCA cycle is derived from glutamine, a proportion comparable to glycolysis and FAO-derived carbon. Glutaminase 1 (GLS1) replenishes glutamine as a carbon source for the TCA cycle, supporting protein and nucleotide synthesis. Additionally, maintaining the dynamic redox balance requires glutathione (GSH), a reducing agent produced from glutamine. Thus, glutamine depletion not only impairs biomass synthesis in ECs but also renders them more susceptible to damage induced by ROS [35]. Silencing GLS1 leads to a reduced tendency for ECs to differentiate into a tip cell phenotype [36]. However, EC-specific deletion of glutamine synthetase (GS), the enzyme that converts glutamate and ammonia to glutamine, markedly impairs EC migration but not proliferation [37]. These findings suggest that glutamine metabolism plays a vital role in regulating the phenotype of ECs during vascular sprouting.

In summary, various metabolic pathways may have significant impacts on the for-mation of different subtypes of ECs. Multiple studies have shown that The level of glycolysis mainly affects the ability of ECs to differentiate towards tip cells, while FA metabolism is closely related to the proliferation of stalk cells. Additionally, glutamine metabolism differentially affects the phenotype of ECs under different conditions.

## Glucose metabolism characteristics of tumor endothelial cells

### Hypoxia of the tumor microenvironment

Hypoxia is one of the characteristics of the tumor microenvironment where neovascularization is usually exposed to relatively low oxygen, and tumor endothelial cells (TECs) need to adjust their metabolism to adapt to the hypoxic tumor environment [38, 39]. In general, cancer cells are characterized by their high metabolism, thus stimulating angiogenesis to supply oxygen and nutrients; however, unlike normal vascular endothelium, blood vessels in tumors are not only disorganized due to abnormal development but also have structural and functional defects due to the lack of tissue hierarchy of normal vascular beds [1]. As a result, the tumor has uneven gaps between vascular endothelial cells, and poor perfusion makes the tumor hypoxic, thereby promoting metastatic escape of cancer cells [40, 41]. In turn, the hypoxic environment stimulates EC proliferation and angiogenesis [42, 43], which creates a vicious cycle of hypoxia and tumor development.

The effects of hypoxia on cancer cell metabolism have become clearer. Briefly, restricted and intermittent blood supply in tumor tissues leads to periodic hypoxia and re-oxygenation, resulting in oxidative stress and hypoxia in the tumor microenvironment. Hypoxic cancer cell metabolism under hypoxic conditions upregulates the expression of genes that promote glycolysis, such as GLUT1, GLUT3, PFKFB3, and HK1-3, while it decreases FAO, promotes FA synthesis, and decreases glutamine carboxylation; all of which contribute to increased glycolytic flux and FA synthesis while downregulating aerobic respiration in hypoxic cancer cells. However, the role of hypoxia and HIF signaling in EC metabolism is still not fully elucidated [44].

Classification of hypoxic conditions has identified acute hypoxia as selective splicing and upregulation of genes involved in pyruvate metabolism and glucose transport [45]. In contrast, the effect of chronic hypoxia on EC metabolism involves the regulation of the activity of multiple metabolic pathways, such as glycolysis, amino acid biosynthesis, carbon metabolism, PPP, and cysteine/methionine metabolism. However, this chronic hypoxia may be different from the intermittent hypoxic conditions of the tumor hypoxic response described previously. Therefore, the changes in EC metabolism observed during chronic hypoxia need to be confirmed in an intermittent hypoxic environment.

### Hypoxia and HIF signaling in angiogenic function of TECs

The prolyl hydroxylase family (PHD) hydroxylates the alpha subunit of HIF. The enzymatic activity of PHD requires oxygen for activation, and the hydroxylated HIF alpha subunit is targeted by the von Hippel‒Lindau (VHL) factor and undergoes ubiquitination and proteasomal degradation [46]. In a hypoxic environment, the enzymatic activity of PHD is inhibited, and VHL factors are unable to degrade HIF-α, leading to the activation of HIF-related pathways and functions, including the induction of angiogenesis, glucose metabolism, and tumor growth, invasion, and metastasis [43]. Among the HIFα subunit family, HIF-1α remains in a stable state during acute hypoxia and is the major subunit that induces glycolysis and other metabolic changes [47].

By focusing on HIF signaling in the study of the tumor hypoxic microenvironment, it has been found that HIF directly induces an increase in VEGF levels in ECs [48], VEGF contains a hypoxia response element (HRE) that can be activated in response to reduced oxygen, causing hypoxic tissues and macrophages to express VEGF and stimulate angiogenesis to restore oxygen and nutrient supply to hypoxic regions. VEGF directly promotes angiogenesis while inducing subsequent activation of other pro-angiogenic growth factors, such as placental growth factor (PlGF) and FGF [49]. Thus, the induction of angiogenesis by hypoxia and HIF is largely dependent on VEGF. Hypoxia and HIF signaling affect EC function and angiogenesis in multiple ways. First, hypoxia induces transcriptional activation of multiple angiogenic factors, including VEGF, PlGF, and angiopoietin (ANGPT1 and ANGPT2) [50]. These vascular growth factors modulate proangiogenic chemokines and receptors that induce EC stem cells to migrate to angiogenic sites [51]. In addition, hypoxia stabilizes these vascular growth factors and promotes PFKFB3 expression, thereby increasing glycolytic flux in ECs [12]. Hypoxia also directly stimulates EC proliferation and neovascular germination as well as promotes neovascular stabilization by remodeling the extracellular matrix [48, 52–54]. In addition, hypoxia and HIF signaling also modulate vascular function by increasing the production and release of NO to promote vasodilation [55].

**Fig. 1:**
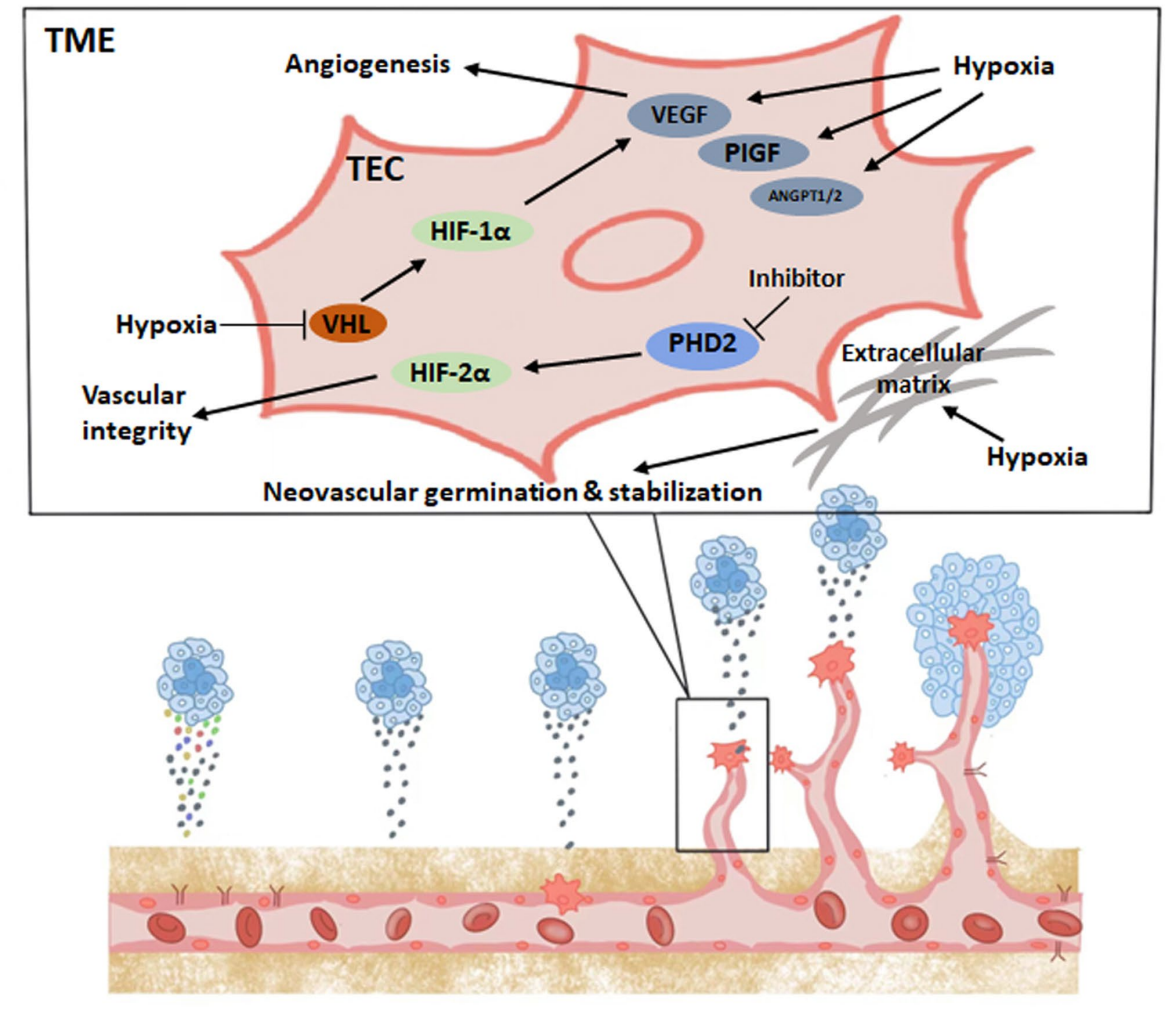
Hypoxia and HIF signaling in angiogenic function of TECs

Stabilization of HIF-1α and HIF-2α has different regulatory effects on angiogenesis and normalization of blood vessels. Specifically, HIF1α deficiency in the tumor endothelium reduces the number of tumor vessels, which reduces tumor growth but increases tumor necrosis; roxarsenical (Rox), an organoarsenic compound, has a significant pro-tumor effect in vivo due to its ability to increase HIF-1α [56, 57]. In contrast, the lack of tumor endothelial HIF2α leads to reduced vascular integrity in tumor models, increasing the likelihood of tumor metastasis but reducing tumor growth [58, 59]. Functional and structural damage to the tumor vasculature increases the likelihood of metastasis, and poor local blood supply also hinders the full efficacy of chemotherapy and immunotherapy. Defective PHD2 mediates the stabilization of HIF2α in ECs, normalizes TECs, reverses tumor hypoxia, and reduces cancer cell metastasis [60]. Here, we draw a schematic diagram to summarize this chapter. (Fig. 1). Thus, targeting the PHD2-HIF2α signaling pathway may be a future tumor treatment strategy based on EC metabolism with antiangiogenesis as the goal.

### Glycolytic characteristics of TECs

Endothelial cells in tumor vasculature have higher glycolytic flux to produce ATP compared to normal endothelial cells, and single-cell RNA sequencing has shown that the glycolytic flux of TECs is 2–4 times higher than that of normal ECs [61]. TECs exhibit a highly glycolytic cellular phenotype, such as enhanced expression of the GLUT1 glucose transporter and the PFKFB3 glycolytic activator [62, 63], which may be attributed to the hypoxia-dependent altered expression of glycolytic enzymes and increased secretion of proangiogenic growth factors, such as VEGF. In response to the high glycolytic phenotype of TECs, the hypoxic tumor microenvironment (TME) and the stimulation of inflammatory factors may be critical for the upregulation of PKFBF3 expression [63]. Reducing the glycolysis of tumor endothelial cells by inhibiting the function of PFKFB3 inhibits their proliferation and thus normalizes the tumor vasculature as evidenced by regular TEC alignment, a dense tumor vascular barrier, and unobstructed blood perfusion. The aim of this therapeutic strategy is not to completely remove the glycolytic flux, which would lead to endothelial cell death and tumor vascular disintegration, but rather to reduce the glycolytic level to that of normal endothelial cells, inhibit their metastasis, and prevent the development of advanced cancer [64, 65]. In addition, the pentose phosphate and serine biosynthetic pathways for biosynthesis are highly activated in tumor endothelial cells compared to healthy endothelial cells [63].

The expression of PKM2, one of the key enzymes of the glycolytic pathway, is traditionally thought to be associated with the proliferation of endothelial cells, especially TECs; however, it has been shown that PKM2 is not required for EC proliferation as PKM2-deficient ECs do not significantly differ from controls in vitro or in vivo, and cell cycle arrest in the absence of PKM2 may be driven by compensatory PKM1 upregulation [17]. PKM2 accumulates at the junctions of VE-cadherin-expressing endothelial cells and the area near F-actin-rich filopodium and lamellipodium, and VE-cadherin is a major regulator of EC junction formation and stability. After knockdown of PKM2, ECs exhibit unstable intercellular junctions and decreased migration distance, demonstrating that the migration ability of ECs and intercellular junction ability are related to the expression of PKM2, which is closely related to the migration ability of ECs and intercellular connectivity. Although the importance of the glycolytic pathway in maintaining EC function and normalizing TECs is well established, only the PFKBF3 glycolytic enzyme has been shown to regulate inter-EC connections by some unknown mechanism [63]. In addition to glycolytic enzymes, extracellular vesicles (H-EVs) have recently been found to promote adhesion of cancer cells to endothelial cells in triple-negative breast cancer, and Circulating galectin-3(cirGal-3) enhances this proadhesive effect by a mechanism that may be related to cirGal-3-induced increased expression of ICAM-1, leading to upregulation of glycolysis in endothelial cells [66]. This special function of PKM2 in maintaining the integrity of the endothelial barrier during migration and thus reducing tumor metastasis provides a new therapeutic strategy for antitumor treatment, namely, targeting PKM2 to inhibit tumor metastasis [67].

As mentioned previously, the PPP is one of the bypasses of glycolysis and achieves redox homeostasis in ECs by synthesizing NADPH, and G6PD is an important rate-limiting enzyme for the PPP. Due to the hypoxic conditions in tumors, high levels of reactive oxygen species (ROS) in the TME stimulate endoplasmic reticulum (ER) stress due to insufficient reduction flux generated by the pentose phosphate pathway (PPP), which activates the IRE-1 and PERK signaling pathways, thereby increasing autophagy, while in turn, the PPP can also play a role in autophagy regulation due to changes in G6PD activity [68]. ROS play a role in cancer suppression, but it has been shown that autophagy promotes tumor angiogenesis by activating the JAK2/STAT3 pathway and targeting VEGF; in addition, autophagy regulates the ability of cancer stem cells (CSCs) to differentiate into TECs [69–72].

Although the glycolytic processes in TEC proliferation and tumor angiogenesis are important, it has recently been reported that microvascular endothelial cells may not be glucose-dependent in their early growth phase as they exhibit good growth capacity even in a glucose-deficient environment. Evidence suggests that glutamine metabolism plays a more important role in the early growth of microvascular endothelial cells [73]. This phenomenon suggests that the metabolism of TECs may be different from one species to another, and tumor treatment strategies targeting TEC metabolism may need to change accordingly with their different metabolic profiles.

### Lactic acid metabolism

In addition to having a direct effect on ECs, the hypoxic microenvironment of tumors can also lead to EC uptake of lactate through lactate accumulation, and the effect of lactate on TECs varies. First, TECs increase the expression of lactate dehydrogenase B, which converts lactate taken up by TECs into pyruvate to enter the TCA cycle to promote biosynthesis and energy supply, thus promoting TEC proliferation and angiogenesis. Second, lactate uptake by ECs induces ROS-mediated activation of the NF-kappaB/IL-8 pathway, promoting angiogenesis [74]. In addition, lactate directly regulates tyrosine kinase receptors in ECs and stabilizes N-Myc downstream regulatory gene 3 (NDRG3), thus promoting angiogenesis under hypoxic conditions [75, 76]. Finally, lactate, as a signaling molecule, also enhances angiogenesis by activating the HIF-1a and PI3K/AKT pathways, thereby promoting angiogenesis [75, 77, 78]. Overall, lactate independently stimulates angiogenesis, and lactate accumulation is associated with disease progression in tumors.

Although the acidic environment due to high lactic acid is not conducive to cell survival, TECs rapidly proliferate in a high lactic acid environment, allowing tumor angiogenesis and survival. The reason for this phenomenon is that TECs upregulate the expression of carbonic anhydrase 2 (CAII). Knockdown of CAII reduces the survival of TECs under lactic acidosis and nutrient-adequate conditions [79]. In normal ECs, vascular endothelial growth factor A (VEGFA) induces CAII expression, which is an indirect proangiogenic mechanism of VEGFA. Interestingly, the carbonic anhydrase inhibitor, acetazolamide, does not significantly reduce tumor angiogenesis but instead promotes vascular maturation in tumors, thereby reducing metastasis [79].

Monocarboxylate transporter (MCT) protein, as a lactate transporter protein, is responsible for transporting lactate inside and outside the cell. MCT4 is the predominant regulator of lactate transport in tumor cells and endothelial cells, which are high glycolytic donors; MCT4 promotes EC migration as well as tumor proliferation and invasion under tumor cell and EC coculture conditions, while the MCT-specific blocker, 7ACC1, reverses the tumor-promoting effect of MCT. However, these findings have only been demonstrated in vitro [80].

### Interactions between different cell types in TME

Based on recent studies, we understand that the level of glycolysis in TECs is inter-cellularly regulated by other cell types in the tumor microenvironment as demonstrated by a 2016 report on tumor-associated macrophages (TAMs) affecting EC glycolytic function. Hypoxia upregulates the expression of regulated in development and DNA damage responses-1 (REDD1), which is an inhibitor of mTOR activation. Knockdown of REDD1 in mice results in an mTOR-dependent enhancement of glycolysis levels in TAMs, and competition between TAMs and TECs for extracellular glucose results in a reduction in glycolytic flux in TECs, indirectly promoting normalization of tumor vasculature [81]. Because HIF1α deficiency reduces CXCL1-mediated macrophage recruitment, hypoxia in TECs affects TAM behavior and recruitment, thus altering the interaction between TAMs and TECs in the tumor microenvironment [82].

Cancer associated fibroblasts (CAFs) in the TME maintain a relatively high glycolytic flux in the resting state to maintain basal cell function, and their glycolysis levels are doubled when proliferating [83]. The reason for this is that the activity of PHD proteins in CAFs is inhibited by high levels of ROS from neighboring cancer cells, which subsequently cause autophagic degradation of caveolin-1 by stabilizing HIF-1α. Caveolin-1 is an NO inhibitory protein whose degradation leads to excessive NO production. These high levels of NO lead to mitochondrial dysfunction of CAFs, resulting in the removal of CAFs by mitochondrial autophagy; thus, CAFs require high levels of glycolysis to produce energy and thus supply lactate, which cancer cells use in the TCA cycle along with converted pyruvate for ATP production [84, 85]. CAFs increase glycolytic flux to maintain a specific link with cancer cells. This phenomenon contradicts the mainstream view of the “Warburg effect” in tumors and has been termed the “reverse Warburg effect”. It is reasonable to speculate that highly glycolytic TECs, similar to CAFs, have this special relationship with cancer cells in terms of lactic acid supply.

### TECs preserve functional mitochondria

Although TECs have a highly glycolytic profile similar to that of CAFs, the mitochondria within TECs are not as dysfunctional as those in CAFs; instead, TECs retain functional mitochondria [86]. The majority of pyruvate is converted to lactate at the end of glycolysis and transported out of the TECs and less than 1% of glucose-derived pyruvate is transported to the mitochondria for the subsequent TCA cycle [87]. However, this oxidative phosphorylation process within the mitochondria not only makes the TEC energy supply more flexible but also maintains TEC proliferation and promotes neovascular sprouting by increasing the amount of biosynthesis. Therefore, the oxidative phosphorylation process in mitochondria is crucial for highly proliferating tumor endothelial cells. Although inhibition of mitochondrial respiration induces death of highly proliferating tumor endothelial cells, there is no significant damage to quiescent normal endothelial cells [88–92]. If the oxidative respiratory chain is interrupted by inactivating the ubiqui-none-binding protein, QPC, a subunit of mitochondrial complex III, then amino acid levels in ECs will not be maintained; although the migratory function of ECs is not affected, their ability to proliferate will be greatly reduced, which further emphasizes the importance of mitochondrial function for EC proliferation [93]. This mechanism of cell death through inhibition of mitochondrial respiration may be related to ROS production and uncoupling of mitochondrial membrane potential. Thus, inhibition of mitochondrial function is an antitumor strategy that has not been explored in depth and deserves further investigation.

## Translational implications

Many current targeted antiangiogenic therapies aim to inhibit the VEGF signaling pathway in ECs, which is clinically associated with a high rate of drug resistance. In addition, single antiangiogenic therapies may also increase the potential for tumor metastasis due to hypoxia in the tumor microenvironment [94]. Compared to the genetic stability of normal cells, tumor cells are genetically unstable and have a high mutation rate, making it difficult to advance therapies targeting tumor cell metabolism. It is reasonable to assume that combination therapy would not only benefit the long-term survival of cancer patients but also attenuate the side effects caused by targeted therapies and improve the quality of life. For example, the PFKFB3 inhibitor, PFK15, has been shown to have significant antitumor proliferation and proapoptotic effects in mice [93], and it synergistically inhibits the proliferation and migration of human umbilical vein endothelial cells (HUVECs) when combined with the tyrosine kinase inhibitor, sunitinib [95, 96]. Regrettably, there is little research related to TEC metabolism, and further understanding of the mechanisms involved in the role and effects of TEC glycolysis will facilitate the development of new therapeutic approaches to treat cancer. Here, we present in table form a list of potentially promising therapeutic agents or substances that currently target the TEC glycolytic pathway (Table 1) [63, 64, 97–116].

**Table 1:** Therapeutic potential of glycolytic targeted drugs in human cancers

Name	Target	Cancer type	Status	Reference
Canagliflozin	GLUT	HCC	Clinical phase I/II	[95]
Fasentin	GLUT	Breast cancer	Preclinical	[96]
Phloretin	GLUT	HCC	Preclinical	[97]
Curcumin	GLUT	Fibrosarcoma	Clinical phase I/II	[98]
Methyl jasmonate	Hexokinases	Gastric cancer	Preclinical	[99]
2-DG	Hexokinases	Neuroblastoma, lung cancer, RCC	Clinical phase I/II	[100–102]
Ketoconazole and Posaconazole	Hexokinases	Glioblastoma	Preclinical	[103]
PFK158	PFKFB3	Advanced solid tumor	Clinical phase I	[61]
3PO	PFKFB3	Melanoma, lung cancer, PAAD	Preclinical	[62, 104, 105]
TLN-232/CAP-232	PKM2	RCC	Clinical I/II	[106]
Shikonin	PKM2	colitis-associated colorectal cancer, non-small cell lung cancer, Bladder Cancer, HCC, Ovarian Cancer	Preclinical	[107–111]
PTK787/ZK 222,584 (Vatalanib)	LDH	CRC	Preclinical	[112]
Gossypol	LDH	Prostate cancer, CRC, breast cancer, lung cancer, PAAD, HCC, HNSC	Clinical phase I/II	[113]
Oxamate	LDH	Breast cancer	Preclinical	[114]

*Abbreviations* GLUT, glucose transporter; HCC, hepatocellular carcinoma; 2-DG, 2-deoxy-d-glucose; RCC, renal cell carcinoma; PFKFB3, 6-phosphofructo-2-kinase/fructose-2,6bisphosphatase3; 3PO, 3-(3-pyridinyl)-1-(4-pyridinyl)-2-propen-1-one; PAAD, pancreatic adenocarcinoma; PKM2, pyruvate kinase M2; CRC, Colorectal cancer; LDH, lactate dehydrogenase, HNSC, head and neck cancers

## Conclusions

The regulation of glycolytic metabolism in endothelial cells (ECs) has gained significant attention, particularly in the context of pathological angiogenesis within the tumor microenvironment. This review provides a comprehensive summary of the differences in glycolytic features between normal ECs and tumor endothelial cells (TECs), as well as the factors that affect their regulation. A promising approach for cancer treatment could be targeting the regulation of glycolytic metabolism in TECs, in combination with classical antiangiogenic therapies. Although many agents targeting aerobic glycolysis of ECs are still in the preclinical or early clinical trial stages, with the continuous exploration in this field, it is optimistic that these emerging drugs may provide new options for cancer treatment in the future.

## Data Availability

No datasets were generated or analysed during the current study.
